# Current Nosology of Neural Autoantibody-Associated Dementia

**DOI:** 10.3389/fnagi.2021.711195

**Published:** 2021-07-28

**Authors:** Niels Hansen

**Affiliations:** Department of Psychiatry and Psychotherapy, University Medical Center Göttingen, Göttingen, Germany

**Keywords:** autoimmunity, neural cell-surface autoantibody, nosology, dementia, intracellular antibody

## Abstract

**Background:**

The detection of neural autoantibodies in patients with cognitive decline is an increasingly frequent phenomenon in memory clinics, and demanding as it does a specific diagnostic approach and therapeutic management, it deserves greater attention. It is this review’s aim to present the latest nosology of neural autoantibody-associated dementia.

**Methods:**

A specific literature research via PubMed was conducted to describe the nosology of neural autoantibody-associated dementia.

**Results:**

An autoimmune dementia comprises with an early onset, atypical clinical presentation and rapid progression in conjunction with neural antibodies, signs of inflammation in the cerebrospinal fluid, and a non-neurodegenerative pattern in neuroimaging. An autoimmune dementia is probably present if the patient responds to immunotherapy. Atypical dementia involving neural autoantibodies with mostly N-methyl-D-aspartate receptor antibodies might not fulfill all the autoimmune-dementia criteria, thus it may constitute an independent disease entity. Finally, a neurodegenerative dementia such as the frontotemporal type also coincides with neural autoantibodies such as the subunit ionotropic glutamate receptors 3 of amino-3-hydroxy-5-methyl-4-isoxazolepropionic acid receptor antibodies, dementia with Lewy bodies with myelin oligodendrocytic protein, myelin basic protein antibodies, or Creutzfeldt-Jakob disease with Zic4 or voltage gated potassium channel antibodies. These dementia entities may well overlap in their clinical features and biomarkers, i.e., their neural autoantibodies or neuroimaging patterns.

**Conclusion:**

There are three main forms of neural autoantibody-associated dementia we can distinguish that might also share certain features in their clinical and laboratory presentation. More research is urgently necessary to improve the diagnosis and therapy of these patients, as the progression of their dementia might thus be improved or even reversed.

## Introduction

**FIGURE 1:**
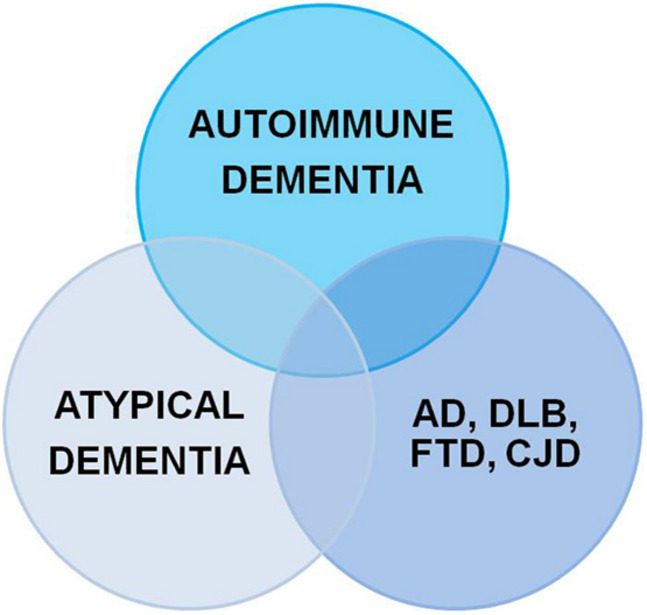
Neural autoantibodies shared by different dementia subtypes. AD, Alzheimer’s disease; DLB, dementia with Lewy bodies; CJD, Creutzfeldt-Jakob disease; FTD, frontotemporal dementia.

Dementia is a serious socioeconomic and medical challenge increasing worldwide. According to the Diagnostic and Statistical Manual of Mental Disorders (DSM-5^®^, fifth edition; American Psychiatric Association, 2013) dementia is defined as an impaired memory function coinciding with other malfunctioning higher cortical functions accompanied by consecutive functional social and occupational impairments. Neural autoantibodies are often associated with cognitive impairment ranging from mild cognitive impairment to dementia (Flanagan et al., 2010; Doss et al., 2014; Gibson et al., 2020; Banks et al., 2021; Hansen et al., 2021a; Timäus et al., 2021). These neural autoantibodies can be classified as autoantibodies against intracellular and membrane-surface antigens. I present below an up-to-date concept for classifying the dementia types associated with neural autoantibodies. Three main types of dementia associated with neural autoantibodies (Figure 1) (and that overlap somewhat among disease entities) are currently distinguished: (1) autoimmune dementia (Flanagan et al., 2010; Banks et al., 2021), (2) atypical dementia (Doss et al., 2014; Gibson et al., 2020), and (3) neurodegenerative dementia (Maetzler et al., 2011; Borroni et al., 2017). The subtypes of neural autoantibody-associated dementia are explained below. Figure 1 condenses the overlap among various dementia disease types with the detection of neural autoantibodies. On the one hand, our finding supports the relevance of neural autoantibodies, pointing toward a specific etiology in one dementia subgroup, but on the other hand, it also confirms the general and not very specific role of neural autoantibodies in these dementia subtypes that might underlie a frequent and potentially relevant immunologic mechanism for disease pathogenesis. The purpose of this review is to provide an overview of the dementia subtypes associated with neural autoantibodies, and how these subtypes might be classified to present a nosology of neural autoantibody-associated dementia.

## Methods

**FIGURE 2:**
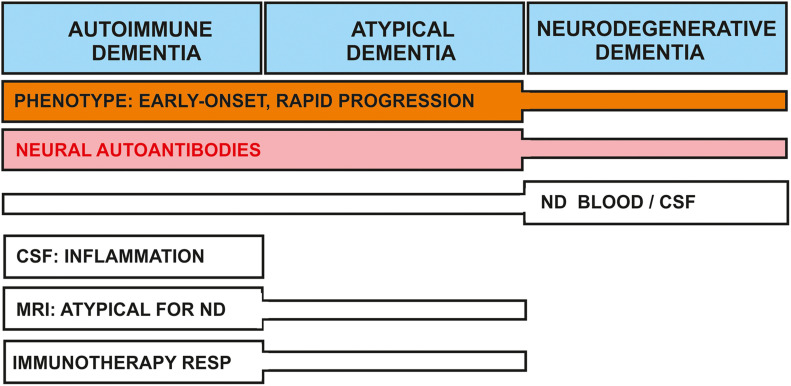
Nosology of dementia associated with neural-autoantibodies: shared and separate features between dementia types. Clinical and laboratory features of dementia subtypes are delineated. The early onset, atypical clinical presentation, and/or rapid progression phenotype is often observed in conjunction with autoimmune and atypical dementias; they are less often encountered in neurodegenerative dementia. Neural autoantibodies may be present in all dementia types, but less frequent in neurodegenerative dementia. Neurodegenerative markers are often positive in neurodegenerative dementia, but are detected less often in autoimmune or atypical dementias. Other features such as CSF inflammation and a non-neurodegenerative pattern in MRI are characteristic for autoimmune and possibly atypical dementia. Patients with autoimmune dementia and occasionally atypical dementia may both be immunotherapy-responsive. ND, neurodegeneration; RESP, response.

For this narrative review, I relied on a PubMed search to identify appropriate articles using the terms “autoimmune dementia,” “dementia and neural autoantibody,” “cognitive impairment and neural autoantibody,” “dementia with Lewy bodies (DLB) and neural autoantibody,” “frontotemporal dementia (FTD) and neural autoantibody,” “Alzheimer’s disease (AD) and neural autoantibody,” “Creutzfeldt-Jakob disease (CJD) and neural autoantibody,” “dementia and autoantibody,” “cognitive impairment and autoantibody,” “DLB and autoantibody,” “FTD and autoantibody,” “AD and autoantibody,” “CJD and autoantibody” in May 2021. The word autoantibody was also replaced by “antibody” in all search terms. As a limitation, no PubMed search was undertaken for other neurodegenerative dementias such as Huntington’s disease, supranuclear palsy or Parkinson’s disease dementia, thus my findings are limited to DLB, AD, CJD, and FTD neurodegenerative dementias. The precipitated results of this narrative review are highlighted in Figure 2.

## Autoimmune Dementia

The latest evidence indicates that autoimmune dementia is an independent and increasingly frequent clinical entity of dementia with certain specific characteristics that must be identified to define this entity. This dementia type is delineated in this review article. The term was coined by Flanagan et al. (2010). Flanagan and his team (Flanagan et al., 2010) refer to a dementia syndrome associated with neural autoantibodies, reveals indices of central nervous system inflammation in cerebrospinal fluid probes, and that is characterized by neuroimaging not typical for a neurodegenerative disease. Furthermore, according to Flanagan et al. (2010) a validated response to immunotherapy is an important element to enable its autoimmune nature to be called probable autoimmune dementia (Flanagan et al., 2010). There are different clinical characteristics that can distinguish autoimmune dementia from a classical neurodegenerative dementia such as AD. The onset age seems to play a major role, as current reports of autoimmune dementia describe the early onset or young age of patients. Furthermore, the clinical course is important, as the cognitive decline in autoimmune dementia deteriorates much faster and more severely than in AD. The difference between an autoimmune encephalitis and dementia depends on the patient’s clinical course: a progressive dementia often suggests an autoimmune dementia, whereas either (1) a relapse-remitting or (2) monophasic manifestation with or without a total or partial reversal of cognitive impairment (Hansen et al., 2018) reflects an autoimmune encephalitis. Finally an atypical clinical presentation revealing as predominant psychiatric features or showing other hints of autoimmune indicators according to Herken and Prüss (2017) might suggest just such an atypical clinical presentation.

### Spectrum of Neural Autoantibodies in Autoimmune Dementia

#### Neural Cell-Surface Autoantibodies in Autoimmune Dementia

There are several known neural antibodies against cell-surface antigens (Table 1) that often coincide with cognitive disturbances (Lai et al., 2009; Hoftberger et al., 2013, 2015; Maat et al., 2015; Gresa-Arribas et al., 2016; Van Sonderen et al., 2016a,b; Hara et al., 2017; Spatola et al., 2017, 2018; Swayne et al., 2018; Brunetti et al., 2019; Zhou et al., 2020; Banks et al., 2021; Hansen et al., 2021a,b; Timäus et al., 2021; Zeng et al., 2021). The most important ones are those against NMDAR, GABABR, and AMPAR, as well as proteins against the cell surface such as LGI1 protein and DPPX, which cognitive dysfunction often accompanies. Young patients often demonstrate autoantibodies against NMDAR. The first neural autoantibodies to be described in association with the term autoimmune dementia were those against voltage gated potassium channels (VGKC), acetylcholine receptors and calcium channels with its subtypes N- or PQ-type (Flanagan et al., 2010). VGKC antibodies are further differentiated as LGI1 and CASPR2 antibodies. VGKC complex antibodies that are not positive for either LGI1 or CASPR2 antibodies are considered double-negative antibodies. These autoantibodies target the cytosolic domain, are thus not termed non-neuronal autoantibodies, and play no relevant pathogenic role in autoimmunity (Van Sonderen et al., 2016a,b; Lang and Prüss, 2017; Lang et al., 2017). In another recent study on patients with dementia, we identified CASPR2, KCNA2 autoantibodies in blood samples (Hansen et al., 2021a). Thus, to date there is a wide spectrum of autoantibodies against cell-surface antigens that are associated with cognitive dysfunction. How these autoantibodies might cause cognitive dysfunction is a matter of debate, and only certain neural cell-surface autoantibodies have been investigated in more depth. The LGI1 antibody is particularly interesting, as it has been investigated applying neuroimaging techniques to elucidate its role autoantibody in cognitive and more specifically memory dysfunction. LGI1 antibodies are detected in patients with specifically bilateral hippocampal subfield atrophy, such as the CA3 hippocampal region in a study by Miller et al. (2020). The hippocampus is the key, pivotal structure forming memory, thus any structural degeneration in its subfields is relevant to memory dysfunction. In fact, bilateral CA3 atrophy resulted in disrupted functional connectivity in resting-state functional magnetic resonance imaging (fMRI) within the temporal lobe default mode network. Furthermore, the disturbances observed in functional temporal connectivity predicted patients’ episodic autobiographic memory performance – indicating the functional relevance of bilateral CA3 atrophy in patients with LGI1 encephalitis. Although autoimmune dementia has not, encephalitis has been reported in conjunction with LGI1 antibodies, and it is conceivable that similar phenomena are related to LGI1 antibodies associated with autoimmune dementia, such as the LGI1 autoantibodies that drive the pathogenesis that might culminate in an autoimmune dementia phenotype. Another study in humans (Loane et al., 2019) emphasized the relevance of LGI1 antibodies in autoimmune brain disease in memory dysfunction terms, as impaired functional connectivity in the hippocampus involving cortical or inter-hippocampal targets correlated with memory performance. These investigations strongly suggest that specific membrane-surface autoantibodies such as LGI1 play a relevant role in orchestrating hippocampus-based memory functions in disease states.

**TABLE 1:** Neural autoantibody-associated dementia and cognitive impairment.

Dementia subtype	Neural autoantibody spectrum	References
**Autoimmune dementia**		
*Cell surface autoantibody*	AChR, AMPAR, CaCh, CASPR2, DPPX, GlycineRalpha1, GABAABR, IgLON5 KCNA2, LGI1, Neurexin 3alpha, NMDAR mGluR5, NMDAR	Lai et al., 2009; Hoftberger et al., 2013, 2015; Maat et al., 2015; Gresa-Arribas et al., 2016; Hara et al., 2017; Spatola et al., 2017, 2018; Swayne et al., 2018; Brunetti et al., 2019; Hansen et al., 2020a; Zhou et al., 2020; Banks et al., 2021; Timäus et al., 2021; Zeng et al., 2021
*Intracellular antibody*	AK5, Amphiphysin, CRMP5/CV2, GAD65 GFAP, Hu, ITPR1, Kelch11, Ma2, Ri, Titin, Yo	Dalmau et al., 1992; Pittock et al., 2005; Honnorat et al., 2009; White and Beringer, 2010; Maat et al., 2015; Do et al., 2017; Ortega Suero et al., 2018; Maudes et al., 2020; Wickel et al., 2020; Budhram et al., 2021; Hansen et al., 2021a; Helmstaedter et al., 2021
**Atypical dementia associated with neural autoantibody**		
*Cell surface autoantibody*	Flotillin 1/2, NMDAR	Doss et al., 2014; Gibson et al., 2020; Hansen et al., 2021b
**Neurodegenerative dementia associated with neural autoantibody**		
*Alzheimer’s dementia*	AChRalpha7, Glia-derived nexin SOS, SP4	Lim et al., 2019; Sim et al., 2019; Goldwaser et al., 2020
*Dementia with Lewy bodies*	MOG, MBP	Maetzler et al., 2011
*Frontotemporal dementia*	AMPAR (GluA3), CaCh (N or PQ type), VGKC	McKeon et al., 2007; Borroni et al., 2017; Younes et al., 2018; Palese et al., 2020
*Creutzfeldt-Jakob disease*	VGKC, Zic4	Jammoul et al., 2014; Salazar, 2018

*AChR, acetylcholine receptor; AK5, adenylate kinase 5; AMPAR, α-amino-3-hydroxy-5-methyl-4-isoxazolepropionic acid receptor; CaCh, calcium channel; CASPR2, contactin-associated protein like 2; CRMP5/CV2, collapsin response mediator protein 5; DPPX, dipeptidyl-peptidase-like protein 6; GABAA/BR, Gamma amino butyric acid A/B receptor; GAD65, glutamic acid decarboxylase 65; GFAP, glial fibrillary acid protein; GluA3, subunit ionotropic glutamate receptors 3; GlycineRalpha1, glycine receptor alpha 1; KCNA2, potassium voltage-gated channel subfamily A member 2; LGI1, leucine rich glioma inactivated protein 1; MBP, myelin basic protein; mGluR5, metabotropic glutamate receptor 5; MOG, myelin oligodendrocytic protein; NMDAR, N-methyl-D-aspartate receptors; VGKC, voltage gated potassium channels.*

#### Neural Intracellular Autoantibodies in Autoimmune Dementia

Several autoantibodies (Table 1) are often now reported to be associated with cognitive dysfunction (Dalmau et al., 1992; Pittock et al., 2005; Honnorat et al., 2009; White and Beringer, 2010; Maat et al., 2015; Do et al., 2017; Ortega Suero et al., 2018; Hansen et al., 2020a,b, 2021a; Maudes et al., 2020; Wickel et al., 2020; Budhram et al., 2021; Helmstaedter et al., 2021). Prominent autoantibody candidates strongly associated with cognitive impairment within the spectrum of autoantibodies against intracellular antigens are those directed against GAD65, AK5, GFAP, and Ma2. Unlike the GAD65 autoantibodies, other autoantibodies are often associated with cancer. In another study of ours, we identified ITPR1, Titin, Yo, and CV2 antibodies in the context of possible autoimmune dementia (Hansen et al., 2021a). Here, it is important to mention that detecting autoantibodies via line blots should be complemented by tissue immunohistochemistry to prevent false-positive results, as demonstrated by Déchelotte et al. (2020) and Ruiz-García et al. (2020). However, the role of intracellular antibodies is not as clear as that of cell-surface autoantibodies in immunopathogenesis, and more research is needed to better understand it. Current evidence has implied cytotoxic CD8+ T-cells (Bien et al., 2012; Ehling et al., 2015; Hansen et al., 2020b) driving the autoimmunity in patients with intracellular autoantibodies.

## Atypical Dementia Associated With Neural Autoantibodies

Another important example of an autoimmune disease in the central nervous system is dementia associated with NMDAR antibodies. NMDAR is important for synaptic transmission within the central nervous system. Studies have shown autoantibodies against NMDAR predominantly in patients presenting atypical early dementia compared to controls. NMDARs are the most thoroughly investigated antibodies that contribute to the pathogenesis of autoimmune disease. NMDAR antibodies (IgG, IgA, and IgM) were detected in 16% of 286 patients with dementia compared to 4% of 47 age-matched controls in a study by Doss et al. (2014; Table 1). Certain clinical characteristics of NMDAR-associated dementia are not typical for neurodegenerative dementia, such as psychiatric features or a history of immune deficiency or cancer (Doss et al., 2014) indicating atypical dementia in these patients. However, some patients with atypical dementia have responded to immunotherapy, i.e., plasma exchange (as revealed in cMRI and PET investigations), thus concurring with a probable autoimmune origin. However, neuroimages taken after immunotherapy have not been systematically assessed in NMDAR-positive atypical dementia, thus the term autoimmune dementia is not fully justified in this context. However, it is important to mention that not only is IgG NMDAR (1.7%) observed – even higher amounts of IgA (4.9%) and IgM (9.5%) antibodies are detected in these patients with dementia. The subgroup of NMDAR antibodies is important to be aware of, as it questions the relevance of NMDAR antibodies in the pathogenicity of dementia, as verified in a recent study with neuronal cultures from Hara et al. (2018) showing that IgG, but not IgA and IgM NMDAR antibodies are very specific to the CNS disease process generating a drop in extrasynaptic and synaptic NMDAR levels. These findings imply that the pathogenic roles played by IgA and IgM are highly questionable, and should be further investigated in dementia patients. Another recent study by Gibson confirmed that both IgG and IgA/IgM NMDAR antibodies might be encountered in patients with atypical dementia (Gibson et al., 2020). In light of the aforementioned study by Hara et al. (2018), the significance of the association between IgA and IgM antibodies and atypical dementia should be enfeebled, and regarded as a probable secondary phenomenon. The meaning of these NMDAR antibody types remains unclear. The atypical nature of these patients’ dementia was characterized by more frequent CSF abnormalities, a rapid onset or fluctuating disease course, a compromised immune reaction such as infections, or coexisting autoimmune disease accompanied by psychiatric features (Doss et al., 2014).

## Neurodegenerative Dementia Associated With Neural Autoantibodies

Neurodegenerative dementia is the most frequent dementia type mainly manifesting as Alzheimer’s dementia. The most frequent is dementia caused by Alzheimer’s disease, which amounts to half of all dementias and is diagnosed by molecular biomarkers according to the ATN classification by Jack et al. (2018). The neuropathological hallmarks of Alzheimer’s disease are the extracellular deposition of β-amyloid peptides and intracellular neurofibrillary bundles composed by aggregated hyperphosphorylated tau protein (Jack et al., 2018). Two other less frequent neurodegenerative dementias are important to mention, namely DLB and FTD, as these have been associated with neural autoantibodies. DLB is the second most frequent dementia, and is characterized by Lewy Bodies; it constitutes approximately 8% of all dementia cases (Walker et al., 2015) and is associated with specific core features such as visual hallucinations, parkinson symptoms or rapid eye movement sleep behavioral disorders, as well as fluctuating cognition according to the McKeith criteria (McKeith et al., 2017). The third most frequent neurodegenerative dementia is FTD; it is characterized by degeneration in the frontal and temporal lobes. Its subtypes reveal mainly behavioral symptoms or speech disturbances according to Gorno-Tempini classification (Gorno-Tempini et al., 2011).

### Frontotemporal Dementia Associated With Neuronal Autoantibodies

#### AMPAR GluA3 Autoantibodies and Frontotemporal Dementia

A recently identified subtype of FTD is FTD associated with the GluA3 subtype of AMPAR antibodies (Borroni et al., 2017; Palese et al., 2020; Table 1). According to a study by Borroni et al. (2017), 41 of 175 (23%) patients with frontotemporal dementia showed antibodies against the AMPAR receptor subunit GluA3. They describe a negative correlation between serum GluA3 antibody titer, and the dementia’s duration in years in their study. AMPAR plays an important role in the synaptic transmission and plasticity that are crucial for learning and memory. The blockage of AMPAR can lead to memory and cognitive dysfunction. Most of the patients presenting GluA3 antibodies in the Borroni et al. (2017) study revealed a presenile-onset, a behavioral variant of frontotemporal dementia, and their MRIs revealed atrophy in bitemporal regions. As other experiments in hippocampal neuron cultures in Borroni’s (Borroni et al., 2017) study with CSF GluA3 autoantibodies demonstrated a loss of GluA3 in AMPAR subunits and elevated tau protein levels, the GluA3 antibodies in these patients are probably relevant for cognitive dysfunction and neurodegeneration.

#### Calcium Channel Autoantibodies and Frontotemporal Dementia

A further antibody target is the P/Q type and N-type calcium channels associated with clinical features of frontotemporal dementia entailing behavioral abnormalities and personality changes (Younes et al., 2018; Table 1) implying the relevance of these autoantibodies in respect to symptoms that concur with the behavioral variant of frontotemporal dementia. The method to detect calcium channel antibodies is important, as the radioimmunoprecipitation assays that used to be (but are no longer) standard procedure bear the risk of false positivity. Thus even healthy subjects might reveal calcium channel antibodies.

#### Potassium Channel Autoantibodies and Frontotemporal Dementia

Moreover, another neural autoantibody was described (McKeon et al., 2007) in conjunction with frontotemporal dementia and speech arrest: they detected antibodies against voltage gated potassium channels (VGKC) in serum (Table 1); immunotherapy resulted in alleviated symptoms, indicating a possible immunotherapeutic effect in some patients with cell-specific autoantibodies such as VGKC presenting the phenotype of frontotemporal dementia.

### Glial Autoantibodies in Dementia With Lewy Bodies

Higher concentrations of antibodies against myelin oligodendrocytic glycoprotein (named MOG) and myelin basic protein (MBP) were detected in conjunction with DLB in serum and CSF compared to other dementias in a study by Maetzler et al. (2011; Table 1). MOG is an adhesion molecule and part of the nerve sheath key to its structural integrity in addition to MBP that also shares an important role in forming the nerve sheath. The increased production of MOG and MBP antibodies in serum and CSF suggest altered activity in DLB’s acquired immune system, making it different from other types of dementia. It is worthwhile investigating whether a psychiatric-onset or MCI-onset of DLB reveals more MOG or MBP autoantibodies than controls to seek novel early biomarkers of DLB. In addition, we are interested to see whether MOG autoantibodies can be detected in those patients suffering from degeneration of the locus coeruleus (LC), as it is the structure that degenerates very early in DLB; some researchers have hypothesized that LC dysfunction might cause the psychiatric phenotype of prodromal DLB (Hansen, 2021). LC degeneration can be detected via neuromelanin-sensitive MR-imaging. Such knowledge could prove relevant to the role of MOG autoantibodies in the initial pathology of DLB and involving LC degeneration, and help us disentangle other psychiatric disorders from a psychiatric-onset of prodromal DLB. Beside the interesting finding of antibodies against glial antigens in DLB and possibly prodromal DLB, the detection method and its results have to be interpreted with caution as cell-based assays are crucial for specificity (Marchionatti et al., 2021); the older-generation assays such as ELISA as in the study by Maetzler et al. (2011) are now considered unreliable and potentially non-specific.

### Neural Autoantibodies in Alzheimer’s Dementia

The deposition of extracellular Aβ is believed to be one of the primordial steps of AD’s pathogenesis (Selkoe and Hardy, 2016). Aβ deposition was shown to be forced by the cross-linking of neuronal alpha7 nicotinic acetylcholine receptor (α7AChR) autoantibody in a recent cell culture model (Goldwaser et al., 2020), thus implying that this neuronal α7AChR autoantibody is associated with the pathogenesis of the most frequent neurodegenerative dementia (Table 1). In recent microarray analysis employing epitope mapping, autoantibodies against peptides were identified and found increased in neuronal cells or pyramidal neurons such as SOS1, or with increased neurofibrillary tangles and neuronal apoptosis such as SP4 (Sim et al., 2019; Table 1). These initial results indicate that autoimmunity against neural cell-surface targets might play an important and so far underestimated role in the pathogenesis of AD. Further research should be implemented to detect novel antibody biomarkers for early AD. In particular, antibodies against glia-derived antigens should be examined, as these are potentially promising biomarker candidates: a glial-derived nexin autoantibody was detected in 80% of AD patients in a study by Lim et al. (2019; Table 1).

Furthermore, a further entity of Alzheimer’s disease should be mentioned that coincides with autoantibodies: β-amyloid-related angiitis. β-amyloid-related angiitis is a subtype of cerebral amyloid angiopathy-related inflammation present with apolipoprotein E ε4 allele and anti-Aβ autoantibodies in CSF (Wu et al., 2021). It is increasingly important to measure and detect such autoantibodies, especially considering the recent United States Food and Drug Administration approval of the anti-amyloid antibody aducanumab, which has the potential side effect of brain swelling and which might be riskier in patients with β-amyloid-related angiitis.

### Neural Autoantibodies in Sporadic Creutzfeldt-Jakob Disease

A patient was recently described (Salazar, 2018; Table 1) presenting subacute symptoms such as mutism, ataxia and rapidly progressing dementia associated with Zic4 antibodies in conjunction with a fulminant disease and 3-month survival. CJD diagnosis was assumedas 14-3-3 protein was elevated and RTQuIC was positive. These autoantibodies might thus be identified in patients presenting a fulminant and rapidly worsening CJD. In addition, Jammoul et al. (2014) reported a case (Table 1) of VGKC antibody in blood plasma coinciding with clinical features of a predominant aphasia and apraxia. Although no clear cognitive decline could be proven neuropsychologically due to the patient’s aphasia, it is nevertheless probable, as their patient seemed very confused and disoriented – which could be related to cognitive decline. This patient’s brain biopsy confirmed CJD through neuropathological investigations via diffuse prion protein-immunostaining employing the 3F4 monoclonal antibody. Genetic analysis (identification of E200K-129M mutation in the gene that encodes the prion protein) confirmed CJD in this patient. Neuronal autoantibodies against membrane-surface antigens were sought in a large cohort study of 346 with suspected and 49 definitive CJD patients (Grau-Rivera et al., 2014). Six of 346 patients with suspected CJD revealed neural surface autoantibodies NMDAR, CASPR2, LGI1, aquaporin 4, Tr [DNER (δ/notch-like epidermal growth factor-related receptor)], although those six patients failed to fulfill the criteria for possible or probable CJD. Positive 14-3-3 proteins were detected in only one patient; a Tr antibody was also detected. Although these findings show that neural autoantibodies in CJD are seldom, such patients should undergo antibody testing to enable a differential diagnosis of autoimmune dementia.

## Association Between Neural Autoantibodies and Neurodegeneration

Neural autoantibody-associated cognitive impairment can damage the brain, as shown by a study by Constantinescu et al. (2016). Their study revealed evidence of reversible changes in axonal degeneration in patients with autoantibody-associated cognitive decline caused by autoimmune encephalitis. Total tau protein and neurofilaments light chain (Nfl), a polypeptide, as markers of axonal degeneration in the acute stage, are elevated, but they gradually decrease later over time. Glial fibrillary acid protein (GFAP) is elevated in both the acute and chronic stage of autoimmune disease. Nfl and tau proteins are markers of acute brain damage; GFAP is also a marker of acute and chronic brain damage in autoimmune encephalitis according to the aforementioned study. In another investigation (Hansen et al., 2021a), we detected autoantibodies against cell-surface and intracellular antigens in 15 of 26 (58%) patients presenting specific autoimmune indicators; we also detected a lower Aβ42/Aβ40 ratio in our patients with biomarker-based Alzheimer’s disease than in those with neural autoantibodies and cognitive impairment. These findings suggest that the pathology of β-amyloid peptides does not play a major role in neural autoantibody-mediated cognitive impairment. However, there has been no systematic investigation yet of whether specific neural cell-surface autoantibodies, such as those against potassium channel Kv1.2 (KCNA2), lead to neurodegeneration. We observed hints of molecular neurodegeneration in KCNA2-associated dementia in a patient series (Timäus et al., 2021) with elevated tau protein and phosphorylated tau protein 181. Our case series’ neuroimaging results vary, revealing mesiotemporal atrophy or unremarkable MRIs, leaving the question open as to whether substantial neurodegeneration might coincide with those findings in autoimmune dementia. A prototypic example of an autoantibody often associated with neurodegeneration is the IgLON5 antibody. A recent investigation demonstrated an interference with IgLON5 antibodies in the neuronal cytoskeleton (Landa et al., 2020), supporting the link between neurodegeneration and autoimmunity. Other studies proved IgLON5 disease and the co-occurrence of axonal brain damage (Hansen et al., 2020a; Landa et al., 2020). However, not just IgLON5 antibodies are known to result in neuroaxonal degeneration, as shown in a study of patients characterized by LGI1, CASPR2 or NMDAR antibodies and encephalitis (Day et al., 2021). However, there has been no prospective study to date examining autoimmune dementia and neurodegeneration. Brain damage can occur in several forms of autoimmune dementia. However, the question remains as to how autoimmune processes trigger neurodegeneration, and whether such neurodegeneration is transient or reversible after immunotherapy.

## Discussion

It is questionable whether these three categories of autoantibody-associated dementia exist as their own categories, or whether they culminate in autoimmune dementia in different phases as the disease progresses. In any case, it is certainly possible that these dementia entities overlap to some degree at different disease stages. More research is required to differentiate these dementia types and how they respond to immunotherapy. The roles of autoantibodies need to be elucidated to be able to differentiate the various types of neural autoantibody-associated dementia. If the autoantibodies are pathogenic as it is believed for antibodies against cell-surface antigens, an autoimmune dementia is more likely, and responds better to immunotherapy. However, T-cell-dependent mechanisms might also contribute to the pathogenesis of autoimmunity in severe cognitive dysfunction, thus autoimmune dementia with intracellular antibodies also exists. We therefore need more animal studies or neuropathological probes to more clearly delineate immune-cell types that might be relevant for disease generation (such as autoreactive T-cells) in order to stratify patients for the most effective therapy.

### Caveats and Limitations

Another limiting factor of the present nosology is that these main subgroups of dementia associated with neural autoantibodies might substantially overlap between disease entities in their clinical presentation and biomarkers, i.e., neural autoantibodies, imaging evidence, or CSF proof of inflammation.

We should highlight a further caveat, namely that some neural autoantibodies are detected at low levels in healthy subjects, so that clues about their significance can only be gleaned from other potential signs of brain damage in such patients. Furthermore, the situation is even harder to interpret in patients with neurodegenerative dementia, as it often accompanied by CSF markers of cell destruction. Thus, the significance of neural autoantibodies in neurodegenerative dementia might be over interpreted. We thus need other biomarkers to more accurately determine whether there is any causal relationship between neural autoantibodies and the neurodegenerative process in neurodegenerative dementia.

In other words, detecting neural autoantibodies in conjunction with neurodegenerative dementia (AD, DLB, PDD, or CJD) might be a secondary, less important phenomenon. The type of neural autoantibody, such as NMDAR IgG compared to NMDAR IgA, or IgM in relation to the patient’s clinical presentation (such as amnestic syndrome and psychosis) might be key to the pathogenicity of neural autoantibodies, and a factor that should be always kept in mind. This distinction is clinically relevant to be able to offer such patients immunotherapy. However, no immunotherapy should be offered yet to those patients presenting a secondary phenomenon (associated with the neural autoantibodies’ unknown pathogenicity) such as in individuals with IgA or IgM NMDAR antibodies revealing no further signs of inflammation in CSF or imaging.

The type of related autoantibody (such as IgLON5) becomes more significant in light of these reflections, and might indicate the need for an immunotherapeutic intervention.

Another factor to consider to distinguish clinically a classic neurodegenerative disorder that will not benefit from immunotherapy from an autoimmune dementia is the clinical disease onset. An early disease onset or rapidly progressing dementia might have an autoimmune etiology in which the neural autoantibodies may play a decisive role in disease pathogenesis (Figure 2).

### Conclusion

In conclusion, neural autoantibody-associated dementia deserves to be better characterized in further investigations, as these dementia subtypes seem to attract less research attention than they warrant; their occurrence in memory clinics is seriously underestimated, as a recent study of neural autoantibody-associated cognitive impairment pointed out (Hansen et al., 2021a). Screening for neural autoantibodies is a useful tool for differential diagnosis (Figure 2), even when a classic neurodegenerative dementia already seems likely.

## Author Contributions

The author confirms being the sole contributor of this work and has approved it for publication.

## Conflict of Interest

The author declares that the research was conducted in the absence of any commercial or financial relationships that could be construed as a potential conflict of interest.

## Publisher’s Note

All claims expressed in this article are solely those of the authors and do not necessarily represent those of their affiliated organizations, or those of the publisher, the editors and the reviewers. Any product that may be evaluated in this article, or claim that may be made by its manufacturer, is not guaranteed or endorsed by the publisher.
